# Recent Advances in the Management of Microangiopathic Hemolytic Anemias (MAHA): A Narrative Review

**DOI:** 10.7759/cureus.47196

**Published:** 2023-10-17

**Authors:** Arundhati Pande, Abhishek Kumar, Harshil Krishnani, Sourya Acharya, Samarth Shukla

**Affiliations:** 1 Medicine, Jawaharlal Nehru Medical College, Datta Meghe Institute of Higher Education and Research, Wardha, IND; 2 Pathology, Jawaharlal Nehru Medical College, Datta Meghe Institute of Higher Education and Research, Wardha, IND

**Keywords:** schistocytes, phagocytosis, arterioles, capillaries, microvasculature

## Abstract

Red blood cells (RBCs) start to break down early in hemolytic anemia, which can be chronic or life-threatening. It should be considered while determining if normocytic or macrocytic anemia is present. Hemolysis in the reticuloendothelial system may happen intravascularly, extravascularly, or both. It accounts for a broad spectrum of laboratory and clinical situations, both physiological and pathological. Whenever the frequency of RBC breakdown is rapid enough to lower hemoglobin levels below the normal range, hemolytic anemia occurs. Microangiopathic hemolytic anemia (MAHA) is a term used to describe non-immune hemolysis induced by intravascular RBC fragmentation caused by substances in the tiny blood arteries that generate schistocytes in the peripheral circulation. Microvasculature abnormalities, such as small arterioles and capillaries, are usually involved. Furthermore, MAHA can also be brought on by intravascular devices like a prosthetic heart valve or assistive technologies. Poor deformity results in entrapment, phagocytosis, antibody-mediated elimination through phagocytosis or direct complement activation, fragmentation brought about by microthrombi or acute mechanical stress, oxidation, or spontaneous cellular death. Hemolysis may cause acute anemia, jaundice, hematuria, dyspnea, tiredness, tachycardia, and possibly hypotension. This article aims to synthesize existing research, identify therapeutic strategies, and provide insights into current and emerging approaches for managing this complex hematological disorder.

## Introduction and background

The two main processes of extravascular hemolysis is brought about by sequestration and phagocytosis [1]. Antibody-mediated hemolysis can occur intravascularly or extravascularly, resulting in phagocytosis and complement-mediated destruction [1,2]. The characteristic of thrombotic microangiopathy (TMA) is microangiopathic hemolytic anemia (MAHA). Red blood cells (RBCs) get harmed within the microvasculature during this process, and thrombocytopenia brought about by platelet activation and consumption also occurs. RBCs inside the peripheral blood are traumatized by intravascular hemolysis caused by strong shear and turbulence in circulation, leading to fragmented RBCs termed schistocytes [3]. Thrombotic thrombocytopenic purpura (TTP) affects around three per 1,000,000 adults, while hemolytic uremic syndrome (HUS) affects approximately three per 100,000 children [4,5]. MAHA is a kind of hemolytic anemia (HA) characterized by erythrocytic fragmentation and hemolysis caused by damage to tiny blood arteries. It may be identified on a blood film by the presence of schistocytes or RBC fragments. Additional symptoms in hemolysis include reticulocytosis, increased lactate dehydrogenase (LDH), low or undetectable haptoglobin, and high unconjugated bilirubin levels [4,5]. However, it is almost always detected as a part of TMA. MAHA may develop as a consequence of a direct impact on RBCs, like damage associated with mechanical heart valves or infections (like malaria) [1,4,6]. MAHA and thrombocytopenia are signs of TMA, which is linked to the formation of thrombi in smaller or larger arteries. In patients having TMA, it is crucial to differentiate between accidental TTP and atypical HUS since they need different treatment plans and prompt management affects the overall prognosis [3]. Sepsis may trigger a disseminated intravascular coagulation (DIC) picture. TMA caused by drugs should be investigated, and any potential causative factors should be eliminated. These conditions are categorized as primary or secondary. Primary TMA (TTP and HUS) develop independently with no known etiology. Following bone marrow transplantation, autoimmune disease, cancer, pregnancy, or even certain medications, secondary types might develop [2,7]. Clinically, the combined presence of MAHA and thrombocytopenia together characterizes TMA syndrome. A histological examination shows micro- and macrovascular thrombosis, with thrombi with varied compositions according to the origin of the TMA [8].

## Review

Search methodology

**Figure 1:**
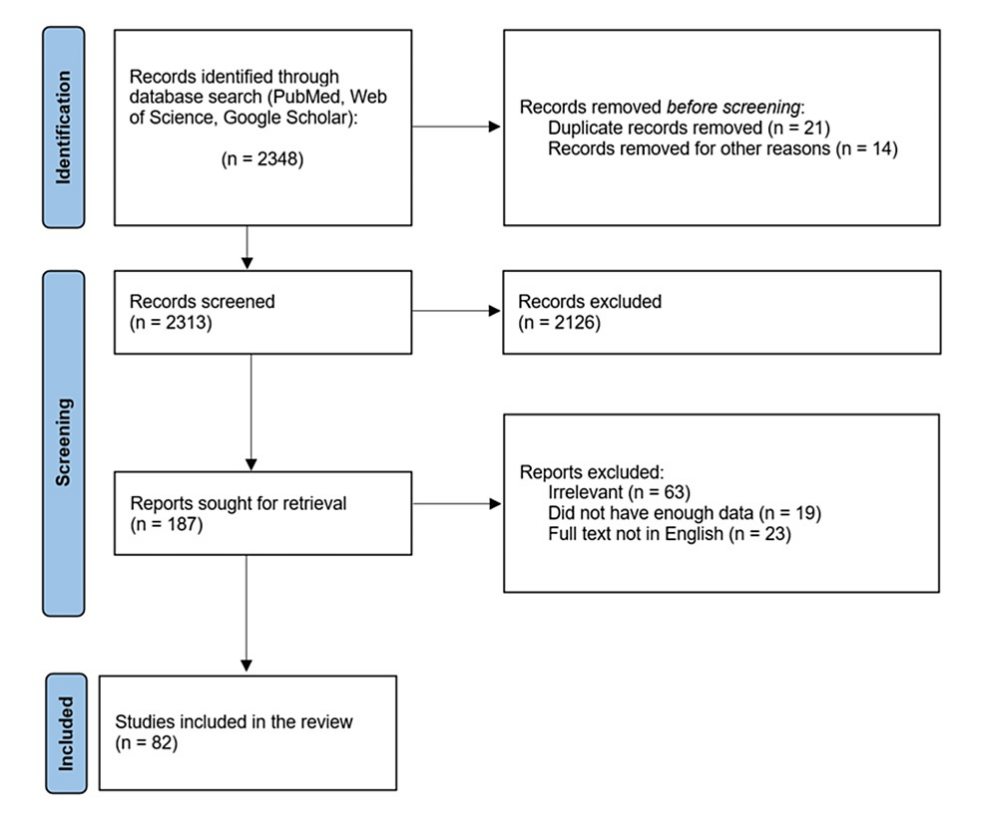
PRISMA flow diagram of the identification process for the sample of 82 articles included in this review. PRISMA: Preferred Reporting Items for Systematic Reviews and Meta-Analysis

The strategies used to make this review include considering research articles published in journals indexed in reputed, reliable, and authentic platforms, processing articles according to different systems, and framing the review like a discussion section of an article where details are explained in straightforward sentences. The databases searched were PubMed, Google Scholar, and Web of Science. Articles published within 20 years were included for review. This review adhered to Preferred Reporting Items for Systematic Reviews and Meta-Analysis (PRISMA) guidelines. Inclusion criteria included microangiopathic causes, while exclusion criteria included malignancy, comorbidities, and renal syndromes. Key terms used for the search are ("hemolysis"[Title/Abstract] OR "hemolytic"[Title/Abstract] OR "erythrolysis"[Title/Abstract] OR "erythrocytolytic"[Title/Abstract]) AND ("microangiopathic"[Title/Abstract] OR "microangiopathy"[Title/Abstract] OR "angiopathic"[Title/Abstract]) AND ("thrombotic"[Title/Abstract] OR "thrombosis"[Title/Abstract] OR "thrombocytopenic"[Title/Abstract]) AND ("anemia"[Title/Abstract] OR (("decrease"[All Fields] OR "decreased"[All Fields] OR "decreases"[All Fields] OR "decreasing"[All Fields]) AND "blood count"[Title/Abstract]) OR "decreased hemoglobin level"[Title/Abstract] OR "hemoglobinemia"[Title/Abstract]). Screening and the number of articles included in the final review are summarized in Figure 1.

MAHA

Extrinsic hemolysis (originating outside) and intrinsic hemolysis (originating inside) are the two types of hemolysis of RBCs [9,10]. Blood collection should be preceded by blood warming to prevent the appearance of spherocytes and erythrocyte agglutination in extravascular hemolysis plus cold agglutinins upon that peripheral smear. The heme oxygenase system in those cells is principally responsible for breaking down hemoglobin. Heme is degraded to bilirubin, while iron is kept and used again, which, on conversion to bilirubin glucuronide in the liver, gets expelled in the bile [11,12]. Extravascular hemolysis occurs when the spleen and liver remove destroyed or dysfunctional RBCs from the circulation. The spleen promotes hemolysis by removing slightly aberrant RBCs or cells with heated antibodies [13,14]. An enlarged spleen can even get blocked with normal RBCs instead of lysed RBCs. RBCs that are severely aberrant or those that are covered with complement (C3) or cold antibodies are killed inside the liver and spleen, which have high blood flow and can quickly remove injured cells [15,16].

**Figure 2:**
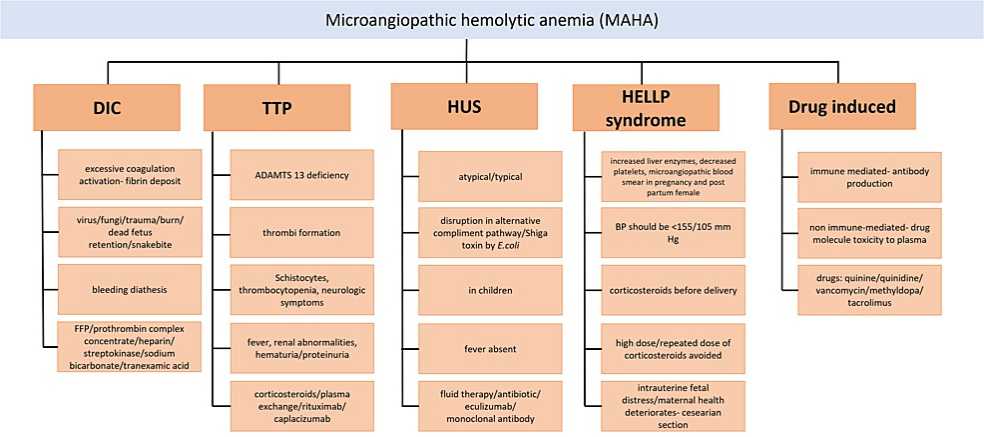
MAHA. MAHA: microangiopathic hemolytic anemia Image Credit: Author

Intravascular hemolysis occurs when the cell membrane is substantially damaged for various causes [17]. Hemoglobinemia results when the amount of hemoglobin released into the plasma exceeds haptoglobin's ability to bind hemoglobin, which is generally present in the plasma at a level of roughly 100 mg/dl (1.0 g/l) [18]. Therefore, less unbound plasma haptoglobin remains present due to intravascular hemolysis. In hemoglobinemia, free hemoglobin dimers are filtered into the urine and subsequently adsorbed by renal tubular cells. When this limit is reached, hemoglobinuria occurs. Iron is released once hemoglobin is catabolized and retained as hemosiderin inside tubular cells. When the tubular cells shed, some iron is excreted in the urine, and some is absorbed for future use [19,20]. RBC fragments (schistocytes) are used to diagnose MAHA upon blood smears. It is hemolysis brought on by RBC destruction from mechanical forces. MAHA is referred to as arteriolar/capillary thrombosis or stenosis in people without intravascular devices such as artificial heart valves, ventricular support devices, or extracorporeal membrane oxygenators. RBC fragmentation is hypothesized to be triggered directly by aberrant shear stress levels brought on by microvascular thrombosis or stenosis [21,22]. Figure 2 shows the classification of MAHA.

DIC

DIC is a common acute or chronic thrombo-hemorrhagic disease in which thrombosis and bleeding are secondary to other illnesses. It may result in microvascular thrombosis, thrombocytopenia, bleeding propensity, and organ failure [23]. It is a clinically and biologically acquired syndrome characterized by excessive coagulation activation, leading to fibrin deposition in the capillaries, organ imbalance, clotting factor, platelet consumption, and potentially fatal hemorrhage [24]. It is a syndrome characterized by excessive thrombin generation within the vasculature and widespread proteolytic conversion to fibrinogen brought on by infectious and non-infectious pathologies like bacterial sepsis, fungus, viruses, trauma, burns, septic abortion, abruption placenta, snake bite, liver conditions, aortic aneurysm, and much more. Regardless of the clinical signs, which might include bleeding diathesis to thrombo-occlusive injury, the pathophysiology of DIC is often linked to excessive blood coagulation activation and concurrent dysregulation of anticoagulants and fibrinolysis. Blood coagulation might start as a result of intravascular expression of a tissue factor or activation of the contact pathway as a result of pathogen or host-derived response to damage in conjunction with molecular patterns. This coagulation is hastened by inflammatory and immune thrombotic processes [25,26]. The most common clinical feature of DIC is bleeding, which is related to tissue hypoxia and infarction brought on by microvascular thrombosis or bleeding diathesis. Clinical and laboratory data must be used to identify DIC. Some of the investigatory findings include reduced platelet count, elevated prothrombin and thrombin times, and the presence of schistocytes in the peripheral smear [27]. Administering these DIC patients fresh frozen plasma (FFP) may be beneficial if they seem to have an extended prothrombin time (PT) as well as an activated partial thromboplastin time (aPTT) [28]. Patients with DIC who are not bleeding are not given prophylactic platelet transfusions unless there is a significant risk of bleeding [29]. If FFP transfusion is still not feasible due to fluid overload in bleeding patients, factor concentrates such as prothrombin complex concentrate should be considered. Therapeutic heparin doses need to be researched in cases of deep vein thrombosis (DVT), including venous and arterial thromboembolism, severe purpura fulminans with acral ischemia, and vascular skin infarction [23,30]. In situations such as venous and arterial thromboembolism, severe purpura fulminans with acral ischemia, or vascular skin infarction, therapeutic heparin dosages should be studied [23,30]. Although typically provoked by underlying illness such as infection or tumor, DIC alone may result in mortality due to bleeding, thrombosis of essential organs, or both. Early detection of acute and life-threatening DIC with proper supporting measures may be lifesaving (Table 1) [31].

**Table 1:** Treatment of DIC. DIC: disseminated intravascular coagulation

Aim of management	Intervention
Control or eradicate the underlying cause, such as removing a dead fetus or placenta [32].	Surgical intervention done for a specific cause.
Precipitating variables such as acidosis, dehydration, sepsis, and hypoxia must be corrected.	Streptokinase and sodium bicarbonate [33-35].
Control bleeding symptoms.	Maintain tissue perfusion and blood volume. This disease is treated using fresh frozen plasma, cryoprecipitates, platelet concentrates, and red blood cell concentrates [36].
Use medications to control coagulation.	Heparin and antifibrinolytics: tranexamic acid. It reduces bleeding episodes by preventing fibrin breakdown by plasmin [37].

TTP

A group of diseases known as TMA, encompassing congenital and acquired etiology, are characterized by thrombosis of the microvasculature and concurrent organ failure. RBC destruction by microvascular thrombi is a hallmark of TMA, leading to HA. In TTP, there is microangiopathic hemolysis, including reduced degradation of von Willebrand factor (vWF) [38,39]. Congenital TTP (cTTP) often manifests in childhood. However, it may also appear later in adulthood. This may happen to women during or shortly after pregnancy. Due to severe ADAMTS13 deficiency, a TMA known as TTP occurs [40]. Normal vWF multimers are secreted into the plasma by endothelial cells and megakaryocytes. These multimers are self-assembled into abnormally large multimers responsible for platelet adhesion. A plasma protease enzyme that modulates the size of multimers and inhibits platelet adhesion is ADAMTS13, a vWF metalloprotease [41]. This shortage may result from a genetic disorder that prevents one from manufacturing enough clipping enzyme or from an overactive immune system that targets and depletes the supply of clipping enzyme [3,42]. As a result, vast multimers of vWF accumulate in the plasma. These multimers activate the spontaneous coagulation cascade, causing platelet binding and the growth of fibrin threads rich in platelets that ultimately form thrombi, causing intravascular hemolysis and ischemic tissue injury [43,44]. The morphology of the peripheral blood smear indicates erythrocyte polychromasia and anisocytosis, as well as abundant schistocytes [2,15]. MAHA with schistocytes (at least three cells per 100), severe thrombocytopenia, temporary neurologic symptoms related to CNS ischemia, fever, and renal abnormalities, including hematuria and proteinuria, are the characteristic signs of TTP [45,46]. Schistocytes and increased serum LDH levels point to TTP [38]. Corticosteroids are added to therapeutic plasma exchange (TPE) for the first acute episode and immune-mediated TTP (iTTP) recurrence. Rituximab is advised; nevertheless, prophylactic TPE is carried out throughout pregnancy for asymptomatic conditions. Prophylactic plasma infusion is indicated for asymptomatic hereditary or cTTP throughout pregnancy, along with a conditional advice for plasma infusion (Table 2) [47-51].

**Table 2:** Treatment of TTP. TTP: thrombotic thrombocytopenic purpura; vWF: von Willebrand factor

Treatment	The action of the regimen
Plasma exchange	Daily plasma exchange involves removing a predetermined quantity of plasma/kg body weight and replacing it with an equal volume of new frozen plasma. It supplies ADAMTS13 and eliminates autoantibodies associated with TTP. Cryoprecipitates and fresh frozen plasma may also be employed [47,48].
Corticosteroids	E.g., prednisolone: it suppresses the antibody formation [49].
Rituximab	A monoclonal antibody that targets CD20 is used. Antibody-producing cells are suppressed [50].
Caplacizumab	Anti-vWF that has been humanized is used to treat TTP. By attacking the A1 domain of vWF, caplacizumab prevents the interaction between vWF and platelets [51].

HUS: typical and atypical

The absence of fever and neurologic symptoms, the prevalence of acute kidney failure (uremia), and the incidence in children separate HUS from TTP. It usually happens when the body is exposed to a specific toxin [52,53]. HUS arises as a result of endothelial damage due to chemicals or medications. *Escherichia coli* (*E. coli*) is the commonest cause of infectious gastroenteritis. It generates a Shiga-like toxin that is absorbed by the irritated gastrointestinal mucosa. This infection must be severe enough to induce bloody diarrhea. The toxin enters the bloodstream and destroys endothelial cells, primarily those in the kidney's glomerular capillaries. Platelets coagulate, thrombi develop, and RBCs rupture due to this [54-56]. Shiga toxin or enterohemorrhagic *E. coli* strain identification from feces is required for diagnosis [57]. Supportive therapy, such as fluid rehydration and RBC transfusions, is often used to treat HUS, along with dialysis when needed [38]. Less frequently, atypical HUS occurs. A disturbance in the alternative complement pathway, which increases complement activity, is the reason. Complement factor H, a crucial regulator of the alternative complement pathway, is the target of autoantibodies which are produced in a limited number of people with atypical HUS [58,59]. The first two criteria for therapy are thrombocytopenia and schistocytic anemia. Plasma exchange should be delivered first to individuals with atypical HUS since the clinical characteristics are often difficult to distinguish from TTP. People with considerable renal insufficiency who do not react to plasma exchange and do not have severe ADAMTS13 deficiency should be suspected of having atypical HUS (Table 3) [2,18].

**Table 5:** Treatment of HUS. HUS: hemolytic uremic syndrome

Treatment	
Supportive care: for renal and hematological complications	When red blood cell transfusion, for example, is used to successfully treat the underlying cause of hemolysis, fluid therapy is often supportive [60].
Antibiotics	Trimethoprim/sulfamethoxazole and lactams have both been associated with an increased risk of HUS. Fluoroquinolones do not seem to worsen the condition despite boosting toxin production in vitro. Fosfomycin and macrolides are the two antibiotics that suppress the production of toxins and may reduce the risk of HUS [61].
For pneumococcal HUS	Eculizumab has been shown to produce a response [61,62].
Experimental	Eculizumab: complement C5 inhibitor [63,64]. Monoclonal antibody to C5 [65,66].

Hemolysis, elevated liver enzymes, and low platelet (HELLP) syndrome and drug-induced TMA

Childbearing age people are susceptible to the illness known as HELLP, which is marked by hemolysis along with a microangiopathic blood smear, increased liver enzymes, as well as low platelets [67-69]. A single course of corticosteroid medication for fetal lung maturation, either two doses of 12 mg betamethasone 24 hours apart or six doses of dexamethasone 12 hours apart, is favoured in gestational ages between 24 and 34 weeks. For fear of long-term harmful effects on the fetal brain, high-dose therapy and repeated dosages should be avoided (Table 4) [70,71]. If the mother's condition deteriorates or intrauterine fetal distress develops prior to 34 weeks of pregnancy, delivery should be performed. Maintain a blood pressure of 155/105 mmHg or less [72,73]. Numerous therapeutic medications may result in thrombocytopenia via immune- or non-immune-mediated mechanisms [74]. Contrarily, immune-mediated thrombocytopenia is brought on by the development of antibodies that react with platelet-specific glycoprotein complexes, as in the case of classic drug-induced immune thrombocytopenia (DITP), or with platelet factor 4, as in the case of heparin-induced thrombocytopenia (HIT) and vaccine-induced immune thrombocytopenia (VITT) [75,76]. A sudden decrease in platelet count, hemorrhage, and thrombosis are all indicators of underlying disease. Because the patient's health might quickly worsen, timely diagnosis and treatment are essential. The most challenging stage in therapy is identifying the substance causing thrombocytopenia [77,78]. Ten to thirteen percent of all instances of TMA and 20-30% of secondary TMA are caused by drug-induced TMA [79]. Some of the drugs accountable include quinine, cyclosporine, tacrolimus, quinidine, penicillin, methyldopa, ticlopidine, clopidogrel, carbamazepine, eptifibatide, ibuprofen, quinine, oxaliplatin, rifampin, sulfamethoxazole, trimethoprim, and vancomycin [80-82].

**Table 6:** Management of HELLP syndrome. HELLP: hemolysis, elevated liver enzymes, and low platelet

Management		Effect
Corticosteroid therapy	Two doses of either 6 mg dexamethasone or 12 mg betamethasone, spaced 24 hours apart, are administered before to birth.	Helps in the lung maturation of the fetus [70,71].

## Conclusions

Early erythrocyte (RBC) destruction in the circulation is the primary cause of HA. Low levels of haptoglobin and hemoglobin, increased reticulocytes, indirect bilirubin, LDH, and characteristic abnormalities on a peripheral blood smear are used for diagnosis. There are many different causes of HA, and each one may lead to the destruction of erythrocytes in different locations, such as larger vessels in the case of autoimmune HA (AIHA) or smaller arteries in the case of MAHA. MAHA is a term used to describe non-immune hemolysis caused by intravascular RBC fragmentation. DIC, HUS, TTP, HELLP syndrome, and consumption of certain drugs are common causes of MAHA. These disorders could be inherited or picked up via the suppression of autoantibodies. TTP is primarily brought on by a marked decline in ADAMTS13's ability to break down the vWF. The standard treatment is plasma exchange in conjunction with corticosteroids. *E. coli* produces Shiga toxin, which causes the normal infection-related HUS. In contrast, complement dysregulation causes atypical HUS. Fluid therapy, antibiotics, and eculizumab are the traditional treatments for HUS. HELLP syndrome is most commonly associated with pregnancy. The choice of treatment involves steroid administration and keeping blood pressure in control. Drug-induced thrombolysis, as the name suggests, is hemolysis caused by specific drug consumption. I suggest you identify that drug and its stoppage or alternative medicine usage. Overall, MAHA's most common clinical features are thrombocytopenia, anemia, jaundice, renal damage, increased bilirubin levels, etc.
